# Supplementary material for: Seminal-Plasma-Mediated Effects on Sperm Performance in Humans
Source: Cells. 2022 Jul 8;11(14):2147. doi: 10.3390/cells11142147 (PMC9322865; doi:10.3390/cells11142147)
Supplement: Supplementary file 1 [file cells-11-02147-s001.zip › cells-1795416-supplementary.pdf]

Supplement table S1. Overall linear mixed model (LMM) statistics for the effect of treatment (control or SP) on VCL and generalized mixed model (GLMM) statistics for the effect of treatment (control or SP) on motility (proportion of motile sperm) and hyperactivation (proportion of hyperactivated sperm). All models included sub-sample, time point, treatment (SW vs. SP), and the interaction between time point and treatment as fixed factors; the random slope of time point for sperm donor, SP donor, and their interaction was included as random effects.

**Effects**		**VCL**				**% of motile sperm**				**% of hyperactivated sperm**			
**Random**			**d.f.**	χ^2	*p*		**d.f.**	χ^2	*p*		**d.f.**	χ^2	*p*
Time point ǀ Sperm donor			2	28.78	**< 0.001**		**3**	**188.35**	**< 0.001**		3	74.56	**< 0.001**
Time point ǀ SP donor			2	30.37	**< 0.001**		**3**	**10.09**	**0.018**		3	58.67	**< 0.001**
Time point ǀ Sperm donor x SP donor			2	9.42	**0.009**		**3**	**10.86**	**0.013**		3	26.64	**< 0.001**
OLRE			-	-	-		1	19.74	**< 0.001**		1	71.03	**< 0.001**
**Fixed**	**Estimate**	**SD**	**d.f.**	**t**	**p**	**Estimate**	**SD**	**z**	**p**	**Estimate**	**SD**	**z**	**p**
Intercept	79.57	5.17	13	15.39	**< 0.001**	-1.03	0.11	-9.46	**< 0.001**	-5.33	0.46	-11.63	**< 0.001**
Sub-sample	-0.66	0.64	591	-1.03	0.301	-0.05	0.02	-3.13	**0.002**	-0.05	0.09	-0.57	0.571
Time point	-13.16	2.52	14	-5.22	**< 0.001**	-0.22	0.04	-5.81	**< 0.001**	-1.92	0.24	-8.02	**< 0.001**
Treatment	42.78	8.45	8	5.06	**< 0.001**	0.19	0.05	4.02	**< 0.001**	2.83	0.93	3.07	**0.002**
Time point x Treatment	16.42	5.58	8	2.94	**< 0.018**	0.22	0.07	2.91	**0.004**	2.04	0.29	6.97	**< 0.001**

Supplement table S2. Ranges of measured sperm parameters at 30 min and 90 min time points in control and seminal plasma-treated samples. VCL = sperm swimming velocity.

Time point	Treatment	Sperm parameter	Range
30 min	Control	VCL (µm s^{-1})	68.1 –161.7
		Motility (%)	16.2 – 76.0
		Hyperactivation (%)	0 – 30.3
	Seminal plasma	VCL (µm s^{-1})	42.8 – 148.9
		Motility (%)	10.9 – 70.7
		Hyperactivation (%)	0 – 16.2
90 min	Control	VCL (µm s^{-1})	64.2 – 172.5
		Motility (%)	13.6 – 70.5
		Hyperactivation (%)	0 – 38.3
		Viability (%)	61.3 – 82.3
	Seminal plasma	VCL (µm s^{-1})	10.9 – 103.3
		Motility (%)	0.9 – 62.5
		Hyperactivation (%)	0 – 4.1
		Viability (%)	66.7 – 86.8

Supplement table S3. Overall linear mixed model (LMM) statistics for sperm curvilinear swimming velocity (VCL) and overall generalized mixed model (GLMM) statistics for motility (proportion of motile sperm) and hyperactivation (proportion of hyperactivated sperm) when considering different SP treatments. All models included sub-sample, timepoint, treatment (own or foreign SP) and its interaction with time point as fixed factors; the random slope of time point for sperm donor, SP donor, and sperm donor-SP donor interaction (random effects).

**Effects**			**VCL**				**% of motile sperm**				**% of hyperactivated sperm**		
**Random**			**d.f.**	χ^2	**p**	**d.f.**		χ^2	**p**	**d.f.**		χ^2	**p**
Time point ǀ Sperm donor			2	43.35	**< 0.001**	3		168.94	**< 0.001**	3		71.60	**< 0.001**
Time point ǀ SP donor			2	37.28	**< 0.001**	3		4.12	**0.030**	3		59.76	**< 0.001**
Time point ǀ Sperm donor x SP donor			2	1.982	0.371	3		8.98	**0.013**	3		2.66	0.448
1 ǀ OLRE			-	-	-	1		11.8	**< 0.001**	1		17.92	**< 0.001**
**Fixed**	**Estimate**	**SD**	**d.f.**	**t**	**p**	**Estimate**	**SD**	**z**	**p**	**Estimate**	**SD**	**z**	**p**
Intercept	80.03	5.09	13	15.72	**< 0.001**	- 1.03	0.11	- 9.36	**< 0.001**	- 5.36	0.53	- 10.08	**< 0.001**
Sub-sample	-1.43	0.61	533	-2.35	**0.019**	- 0.06	0.02	- 3.43	**0.001**	- 0.17	0.12	- 1.41	0.159
Time point	-13.22	2.75	14	-4.81	**< 0.001**	- 0.23	0.04	- 5.70	**< 0.001**	- 2.24	0.33	- 6.77	**< 0.001**
Treatment	0.05	1.71	77	0.03	0.973	-0.02	0.04	-0.63	0.532	-0.11	0.22	-0.51	0.613
Treatment x Time point	1.08	1.97	270	0.55	0.583	0.14	0.06	2.42	**0.016**	0.96	0.45	2.13	**0.033**

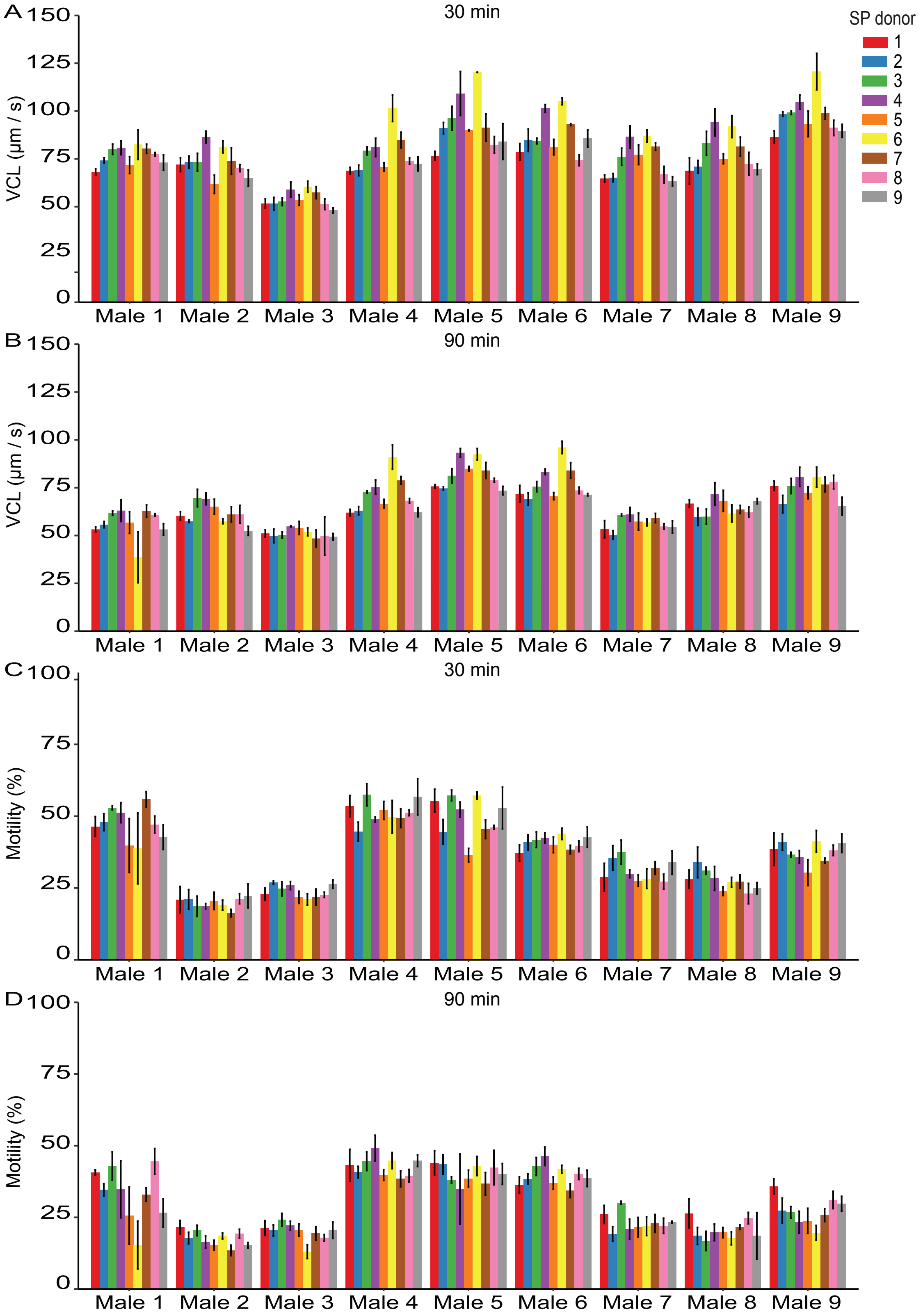

Supplement figure S1. Sperm parameters in SP-treated samples 30 min and 90 min after the treatments: sperm swimming velocity (VCL) at 30min (**A**), VCL at 90 min (**B**), proportion of motile sperm at 30 min (**C**) and proportion of motile sperm at 90min (**D**). Each male (1-9) is represented in separate panels and the SP donor identities are indicated by different colors. Error bars show standard error of mean (SEM).

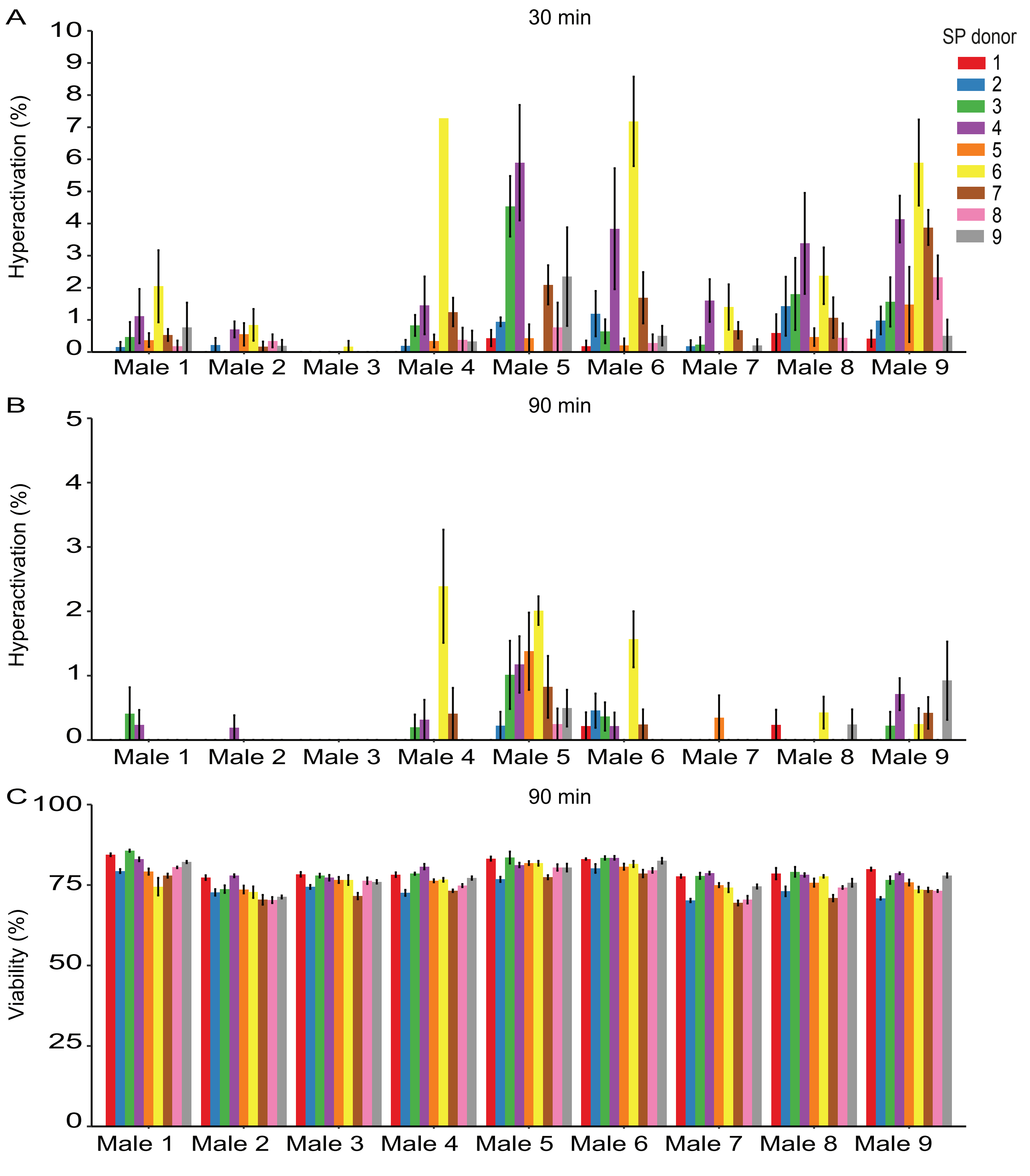

Supplement figure S2. Sperm parameters in SP-treated samples 30 min and 90 min after the treatments: the proportion of hyperactivated sperm at 30min (**A**), at 90 min (**B**) and sperm viability at 90 min (**C**). Each male (1-9) is represented in separate panels and the SP donor identities are indicated by different colors. Error bars show standard error of mean (SEM).
